# Quantum realization of the bilinear interpolation method for NEQR

**DOI:** 10.1038/s41598-017-02575-6

**Published:** 2017-05-31

**Authors:** Ri-Gui Zhou, Wenwen Hu, Ping Fan, Hou Ian

**Affiliations:** 1 College of Information Engineering, Shanghai Maritime University, Shanghai, 201306 China; 2 School of Information Engineering, East China Jiaotong University, Nanchang, Jiangxi 330013 China; 3 Institute of Applied Physics and Materials Engineering, FST, University of Macau, Macau, China

## Abstract

In recent years, quantum image processing is one of the most active fields in quantum computation and quantum information. Image scaling as a kind of image geometric transformation has been widely studied and applied in the classical image processing, however, the quantum version of which does not exist. This paper is concerned with the feasibility of the classical bilinear interpolation based on novel enhanced quantum image representation (NEQR). Firstly, the feasibility of the bilinear interpolation for NEQR is proven. Then the concrete quantum circuits of the bilinear interpolation including scaling up and scaling down for NEQR are given by using the multiply Control-Not operation, special adding one operation, the reverse parallel adder, parallel subtractor, multiplier and division operations. Finally, the complexity analysis of the quantum network circuit based on the basic quantum gates is deduced. Simulation result shows that the scaled-up image using bilinear interpolation is clearer and less distorted than nearest interpolation.

## Introduction

Image is an important medium for visual information transmission. Image processing is very popular because of the need to extract visual information from the natural world. With the rapid development of quantum computation and quantum information in past several decades, quantum computer has demonstrated a bright prospect over the classic computer, such as Feynman’s computation model^1^, Deutsch’s quantum parallelism assertion^2^, Shor’s integer factoring algorithm^3^, and Grover’s database searching algorithm^4^.

Quantum image processing (QIMP), a new sub-discipline of information and image processing, which is devoted to utilizing the quantum computing technologies to capture, manipulate, and recover quantum images in different formats and for different purposes. The investigation of QIMP begins with how to store and retrieve quantum images in quantum computers. Venegas-Andraca and Bose firstly proposed the quantum image representation of qubit lattice using one qubit to hold one pixel^5^. Then Latorre presented real ket representation using quantum superposition state to store image information^6^. Le *et al*.^7^ next proposed a flexible representation of quantum image (FRQI) using quantum superposition state to store the colors and the corresponding positions of an image. Further, more quantum image representations were proposed. For instance, a novel enhance quantum representation (NEQR)^8^ used *q* qubits encoding the gray-scale value from 0 to 2^*q*^ − 1, which could perform the complex and elaborate color operations conveniently. Quantum log-polar image^9^ was proposed as a novel quantum image representation storing images sampled in log-polar coordinates. Color image representation utilized two sets of quantum states to store *M* colors and *N* coordinates, respectively^10^. A normal arbitrary quantum superposition state was used to represent a multi-dimensional image^11^. After that, a simple quantum representation of infrared images was proposed^12^.

In addition, some geometric transformation algorithms of quantum images were designed such as two-point swapping, flip, orthogonal rotations, entire translation, cyclic translation, global and local translation^13–15^. Then, the quantum image scaling algorithms^16–18^ were proposed. In paper^16^, Sang J *et al*. realized quantum image scaling for FRQI and NEQR using nearest-neighbor interpolation method. Jiang N *et al*. designed quantum circuits of quantum image scaling^17^. Furthermore, Jiang N *et al*. proposed the generalized quantum image representation (GQIR) and quantum image scaling up based on GQIR and nearest-neighbor interpolation with integer scaling ratio^18^.

In the aspects of quantum protection, some algorithms have appeared recently such as quantum image scrambling^19–21^, quantum watermarking schemes^22–25^, LSB steganography algorithms based on NEQR^26, 27^.

## Preliminaries

### The novel enhanced quantum representation (NEQR)

The NEQR^8^ is described as follows:

Supposing the range of the gray-scale value is from 0 to 2^*q*^ − 1, the gray-scale value *C*_*YX*_ of the pixel coordinate (*Y*, *X*) can be expressed by Eq. (1).

1 $${C}_{YX}={C}_{YX}^{q-1}{C}_{YX}^{q-2}\cdots {{\rm{C}}}_{{\rm{YX}}}^{1}{{\rm{C}}}_{{\rm{YX}}}^{0},{C}_{YX}^{k}\in \{0,1\},{C}_{YX}\in [0,{2}^{q}-1]$$

Hence, NEQR for a 2^*n*^ × 2^*n*^ quantum image can be written as

2 $$|{\rm{I}}\rangle =\frac{{\rm{1}}}{{{\rm{2}}}^{{\rm{n}}}}\sum _{Y=0}^{{2}^{n}-1}\sum _{X=0}^{{2}^{n}-1}|{C}_{YX}\rangle |Y\rangle |X\rangle =\frac{{\rm{1}}}{{{\rm{2}}}^{{\rm{n}}}}\sum _{YX=0}^{{2}^{2n}-1}\underset{k=0}{\overset{q-1}{\otimes }}|{C}_{YX}^{k}\rangle \otimes |YX\rangle $$

**Figure 1:**
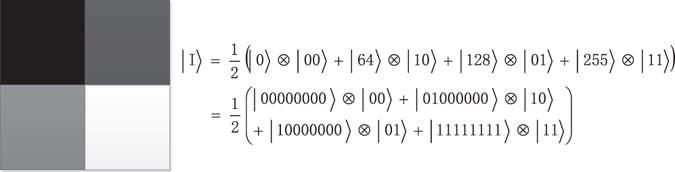
An example of 2 × 2 image and its NEQR.

Figure 1 shows an example of a 2 × 2 image, and the corresponding NEQR of which is on the right.

### Classical bilinear interpolation method

Bilinear interpolation method plays an important role in classical image scaling. In this paper, we mainly study the quantum realization of bilinear interpolation method. Thus, the classical bilinear interpolation method is reviewed.

For a *W* × *H*(width and height) image, the size of the corresponding interpolated image is *W*′ × *H*′, which can be described in two steps.

#### Coordinate map

**Figure 2:**
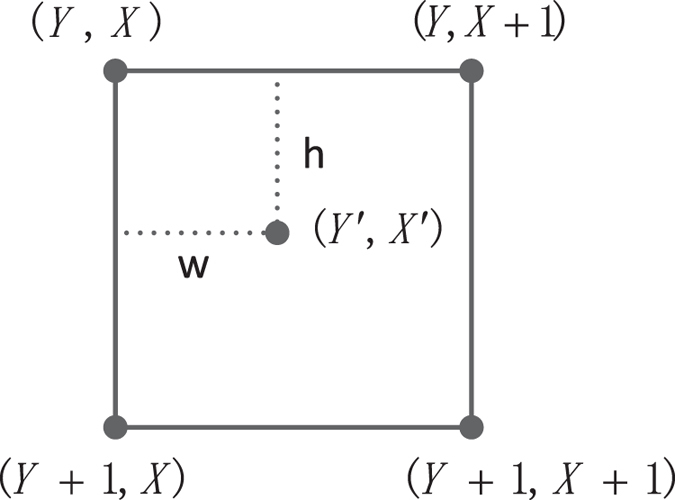
Coordinate map relationship.

The coordinate (*Y*′, *X*′) of the interpolated image is restored from the positions (*Y*, *X*), (*Y* + 1, *X*), (*Y*, *X* + 1) and (*Y* + 1, *X* + 1) in the original image. The corresponding relationship is shown in Fig. 2.

Here,

3 $${\rm{Y}}=\lfloor {\rm{Y}}^{\prime} \times \frac{H}{H^{\prime} }\rfloor ,X=\lfloor X^{\prime} \times \frac{W}{W^{\prime} }\rfloor ,{\rm{h}}=\frac{H}{H^{\prime} }Y^{\prime} -Y,w=\frac{W}{W^{\prime} }X^{\prime} -X$$

#### Calculating pixel value

**Figure 3:**
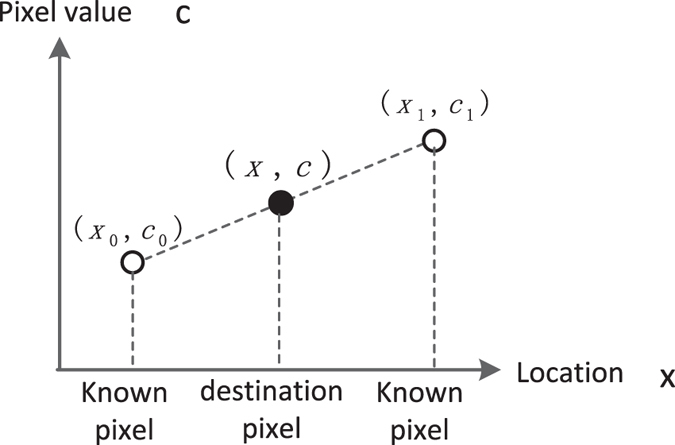
Bilinear interpolation method.

As shown in Fig. 3, the value of the destination pixel (*x*, *c*) can be obtained by Eq. (4)

4 $$\frac{c-{c}_{0}}{x-{x}_{0}}=\frac{{c}_{1}-c}{{x}_{1}-x}$$

where (*x*_0_, *c*_0_) and (*x*_1_, *c*_1_) are two known pixels. That is to say,

$$c=\frac{({x}_{1}-x){c}_{0}+(x-{x}_{0}){c}_{1}}{{x}_{1}-{x}_{0}}$$

An interpolation can be described in Eq. (5)

5 $$I^{\prime} =S(I,{r}_{x},{r}_{y})={S}_{y}({S}_{x}(I,{r}_{x}),{r}_{y})={S}_{x}({S}_{y}(I,{r}_{y}),{r}_{x})\frac{n!}{r!(n-r)!}$$

where *S* is the scaling function, *I* is the original image, *I*′ is the interpolated image, *r*_*y*_ is the scaling ratio in vertical, and *r*_*x*_ is the scaling ratio in horizontal. Thus, the pixel value in position (*Y*′, *X*′) of the interpolated image shown in Fig. 2 can be calculated according to Eq. (6)

6 $$\begin{matrix}f(Y^{\prime} ,X^{\prime} ) & = & (1-h)(1-w)f(Y,X)+h(1-w)f(Y+1,X)\\  &  & +(1-h)wf(Y,X+1)+hwf(Y+1,X+1)\end{matrix}$$

### Quantum circuit realization

In this section, we design a series of quantum circuit modules to realize some special functions.

### Basic reversible quantum gates

There are a number of existing 3 × 3 reversible gates such as Fredkin gate^28^, Toffoli gate^29^, Peres gate^30^ and Thapliyal Ranganathan gate^31^. The quantum cost^32, 33^ of a reversible gate is the number of 1 × 1 and 2 × 2 reversible gates required in its design. The cost of all the 1 × 1 reversible gates is assumed to be zero such as NOT gate, and the cost of all 2 × 2 reversible gates is taken as unity. Any reversible gate can be realized using 1 × 1 NOT gates and 2 × 2 reversible gates such as Controlled-*V*, Controlled-*V* ^+^ and Controlled NOT (CNOT) gates. Thus, in simple terms, the quantum cost of a reversible gate is regarded as the number of NOT, Controlled-*V*, Controlled-*V* ^+^ and CNOT gates required in its implementation circuit. Here, we briefly introduce some basic quantum gates first.

#### The NOT Gate (*X* gate) and Hadamard Gate (*H* gate)

**Figure 4:**
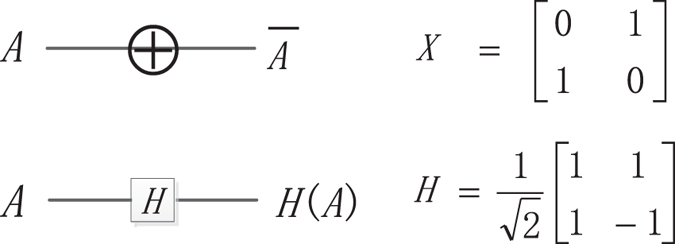
*X* gate and *H* gate.

The symbolic representation and matrix representation of *X* gate and *H* gate are shown in Fig. 4.

The function of *X* gate and *H* gate is as follows:

$$X|0\rangle =|1\rangle ,\,X|1\rangle =|0\rangle $$

and

$$H|0\rangle =\frac{1}{\sqrt{2}}(|0\rangle +|1\rangle ),H|1\rangle =\frac{1}{\sqrt{2}}(|0\rangle -|1\rangle )$$

where $$|0\rangle =[\begin{matrix}1\\ 0\end{matrix}],|1\rangle =[\begin{matrix}0\\ 1\end{matrix}]$$.

#### The Controlled-*V* and Controlled-*V*^+^ Gates

**Figure 5:**
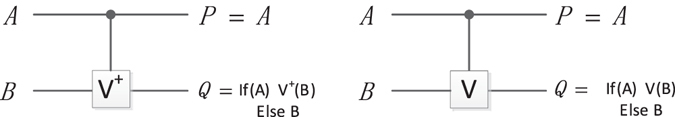
The Controlled-*V* and Controlled-*V* ^+^ gates.

The Controlled-*V* and Controlled-*V* ^+^ gates are shown in Fig. 5. If the control signal *A* = 0, then the qubit *B* will pass through the controlled part unchangeably, i.e., *Q* = *B*. When *A* = 1, then the unitary operation is applied to the input *B*, i.e., *Q* = *V* (*B*) or *V* ^+^ (*B*). Where *V* is a square-root of *X* gate and $$V=\frac{i+1}{2}(\begin{matrix}1 & -i\\ -i & 1\end{matrix})$$.

The *V* and *V* ^+^ quantum gates have the following properties:

$$\begin{matrix}V\times V & = & {V}^{+}\times {V}^{+}=X\\ V\times {V}^{+} & = & {V}^{+}\times V=I\end{matrix}$$

where $$I=[\begin{matrix}1 & 0\\ 0 & 1\end{matrix}]$$ is an identity matrix. More details of the *V* and *V* ^+^ gates refer to the literature^32, 34^.

#### The CNOT gate

**Figure 6:**
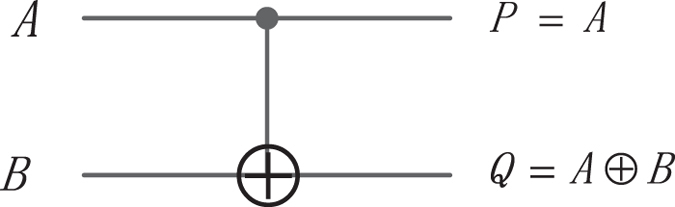
The CNOT gate.

The CNOT gate shown in Fig. 6 has the mapping (*A*, *B*) to (*P* = *A*, *Q* = *A* ⊕ *B*), where *A*, *B* are the inputs and *P*, *Q* are the outputs, respectively.

#### The Toffoli gate (TG)

**Figure 7:**
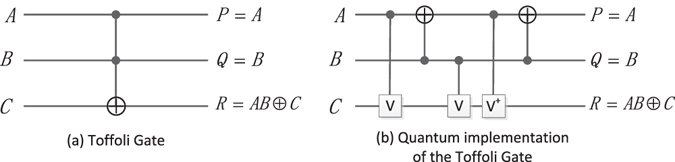
The TG and its quantum circuit.

The TG and its quantum circuit realization are shown in Fig. 7. The quantum cost of TG is 5, which can be seen from Fig. 7(b).

#### The Peres gate (PG)

**Figure 8:**
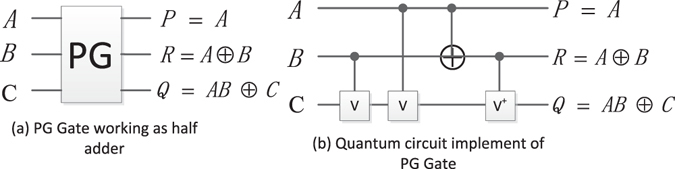
PG and its quantum circuit.

The PG is shown in Fig. 8(a). The quantum circuit of PG is shown in Fig. 8(b), then, we can get that the quantum cost of PG is 4.

#### The Thapliyal Ranganathan gate (TR)

**Figure 9:**
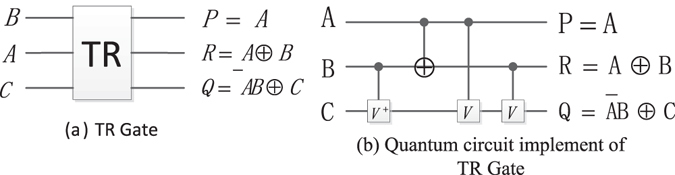
TR gate and its quantum circuit.

TR gate and its quantum circuit are shown in Fig. 9. The quantum cost of TR gate is also 4.

### Special adding one operation

**Figure 10:**
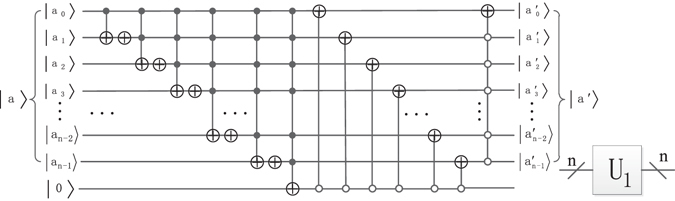
Quantum circuit of U_1_ (n) module and its simplified graph.

The special adding one operation *U*_1_ (*n*) module is shown in Fig. 10, where the label • and ◦ represent the control qubit value $|{\rm{1}}\rangle$ and $|{\rm{0}}\rangle$, respectively. When *U*_1_ (*n*) works on the quantum state $|{a}_{n-1}{a}_{n-2}\cdots {a}_{1}{a}_{0}\rangle$, then the result is

$${U}_{1}(n)|{a}_{n-1}\cdots {a}_{1}{a}_{0}\rangle =\{\begin{matrix}|{a}_{n-1}\cdots {a}_{1}{a}_{0}+1\rangle ,\,{a}_{n-1}\times {a}_{n-2}\times \cdots \times {a}_{1}\times {a}_{0}\ne \{0,1\}\\ |{a}_{n-1}\cdots {a}_{1}{a}_{0}\rangle ,\,{a}_{n-1}\times {a}_{n-2}\times \cdots \times {a}_{1}\times {a}_{0}=\{0,1\}\end{matrix}$$

where *n* is a positive natural number, *n* ≥ 2, *a*_0_, *a*_1_, …, *a*_*n*−1_ ∈ {0, 1}_._

### The multiply Control-Not operation

**Figure 11:**
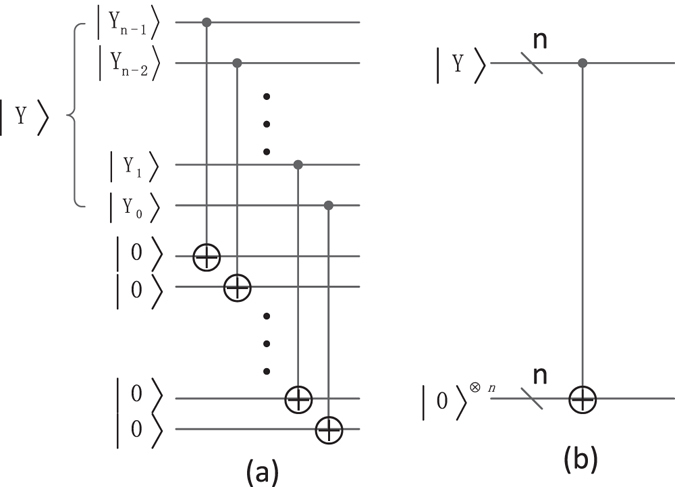
Multiple Control-Not operation and its simplified graph.

The quantum circuit of the multiply Control-Not operation is shown in Fig. 11(a) and its simplified graph is shown in Fig. 11(b). It utilizes *n* Control-Not gates to copy the *n*-qubit information of $|Y\rangle =|{Y}_{n-1}{Y}_{n-2}\cdots {Y}_{1}{Y}_{0}\rangle$ into the *n* ancillary qubits ${|0\rangle }^{\otimes n}$, where $|{Y}_{n-1}\rangle ,|{Y}_{n-2}\rangle ,\cdot \cdot \cdot ,|{Y}_{1}\rangle$ and |*Y*_0_〉 are the control qubits and the *n* ancillary qubits ${|0\rangle }^{\otimes n}$ are the target qubits. That is, the input $|Y\rangle \otimes {|0\rangle }^{\otimes n}$ is changed into the output $|Y\rangle \otimes |Y\rangle$ by using the multiply Control-Not operation.

### The reversible parallel full-adder circuit

Islam M S *et al*.^35^ proposed the reversible full-adder based on the PG. Here, the introduction of the design of half-adder, full-adder and parallel-adder are given.

#### Reversible half adder (RHA)

**Figure 12:**
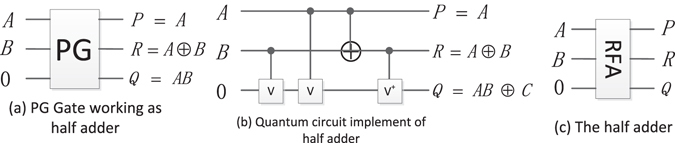
The half-adder and its quantum circuit.

Figure 12 shows the PG working as a half-adder and its quantum circuit, where *R* = *A* ⊕ *B* represents the sum of *A* + *B* and *Q* = *AB* represents the carry, respectively.

#### Reversible full adder (RFA)

**Figure 13:**
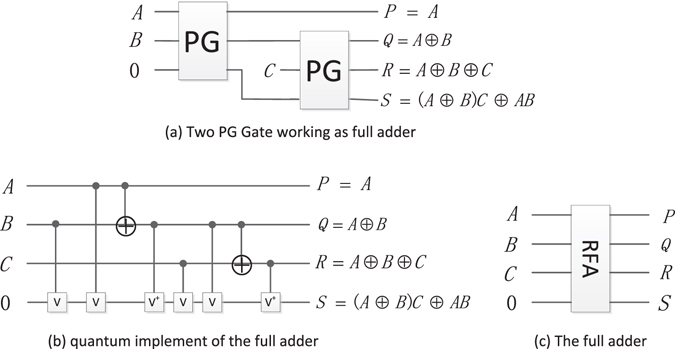
The full-adder and its quantum circuit.

Using two PG gates, the full-adder can be designed shown in Fig. 13(a), where *R* = *A* ⊕ *B* ⊕ *C* represents the sum of (*A* + B + C) and *S* = (*A* ⊕ *B*)*C* ⊕ *AB* represents the carry, respectively.

The quantum circuit of RFA is shown in Fig. 13(b), and its simplified graph is shown in Fig. 13(c).

#### Reversible parallel adder (PA)

**Figure 14:**
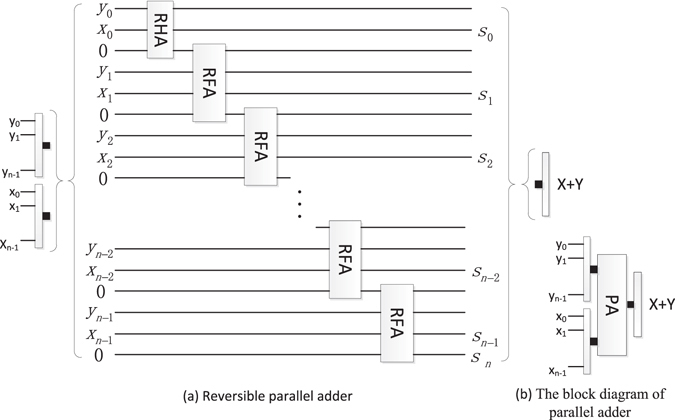
The reversible parallel adder and its block diagram.

The parallel adder adding an *n*-qubit *Y* to an n-qubit *X* is designed by one RHA and *n*-1 reversible full-adders as shown in Fig. 14. Here, the sequence ${S}_{n}{S}_{n-1}\cdots {S}_{1}{S}_{0}$ represents the sum of X + Y. Other unremarked qubits are the garbage outputs and the input qubit 0 is the ancillary constant input. For convenience, the block diagram of PA omits the ancillary inputs and the garbage outputs.

### The reversible parallel subtractor circuit

Thapliyal H. *et al*.^31^ designed subtractor using the reversible TR gate and further realized optimization in terms of quantum cost and delay^36^. Here, the concrete parallel subtractor circuit is given.

#### Reversible half subtractor (RHS)

**Figure 15:**
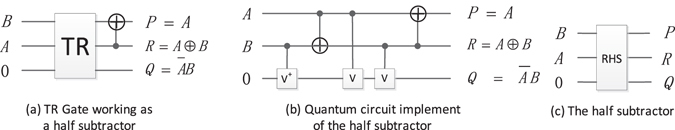
The RHS and its quantum circuit.

As shown in Fig.15, the inputs of *A* and *B* are 1-bit binary number, and the TR gate can work as a half subtractor performing *A*-*B* operation, where the output *R* = *A* ⊕ *B* produces the difference between *A* and *B* and the output $Q=\overline{A}B$ generates the corresponding borrow bit. The quantum circuit of RHS is shown in Fig. 15(b), and its simplified graph is shown in Fig. 15(c).

#### Reversible full subtractor (RFS)

**Figure 16:**
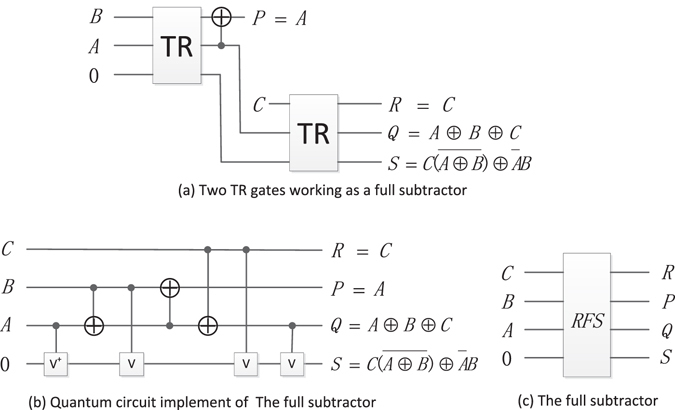
The RFS and its quantum circuit.

The RFS as shown in Fig. 16 is utilized to realize the operation *Y* = *A* − *B* − *C*, where *Q* = *A* ⊕ *B* ⊕ *C* represents the difference of *A* − *B* − *C*, $S=C(\overline{A\oplus B})\oplus \overline{A}B$ represents the borrow bit. The quantum circuit of RFS is shown in Fig. 16(b), and its simplified graph is shown in Fig. 16(c).

#### Reversible parallel subtractor (PS)

**Figure 17:**
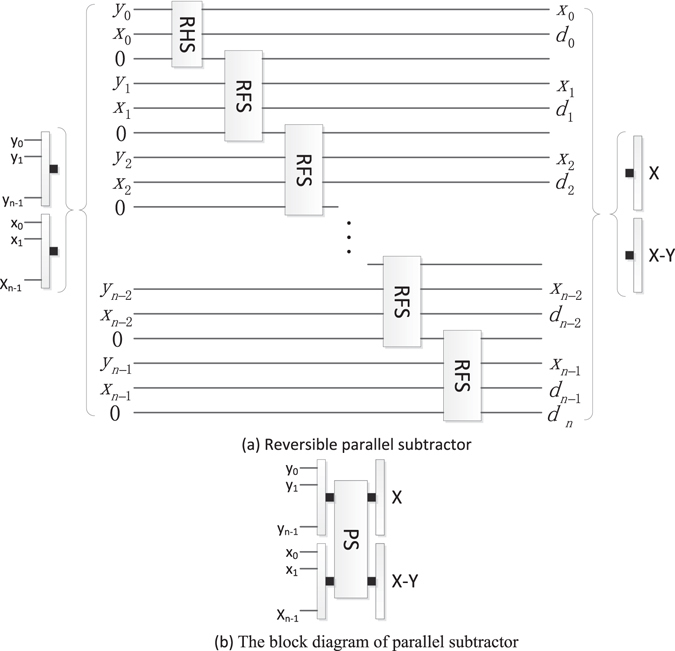
The PS and its block diagram.

The PS is used to compute the difference of two *n*-bit numbers *X* and *Y*, where $X={x}_{n-1}\ldots {x}_{0}$ and $Y={y}_{n-1}\ldots {y}_{0}$. The PS subtracting an n-qubit *Y* from a n-qubit *X* is designed by one RHS and *n*-1 reversible full subtractors as shown in Fig. 17, where ${d}_{n}{d}_{n-1}\ldots {d}_{1}{d}_{0}$ is the result of *X*-*Y*, and the ancillary constant input is 0. For convenience, the block diagram of PS omits the garbage outputs.

### The reversible parallel multiplier (PM)

**Figure 18:**
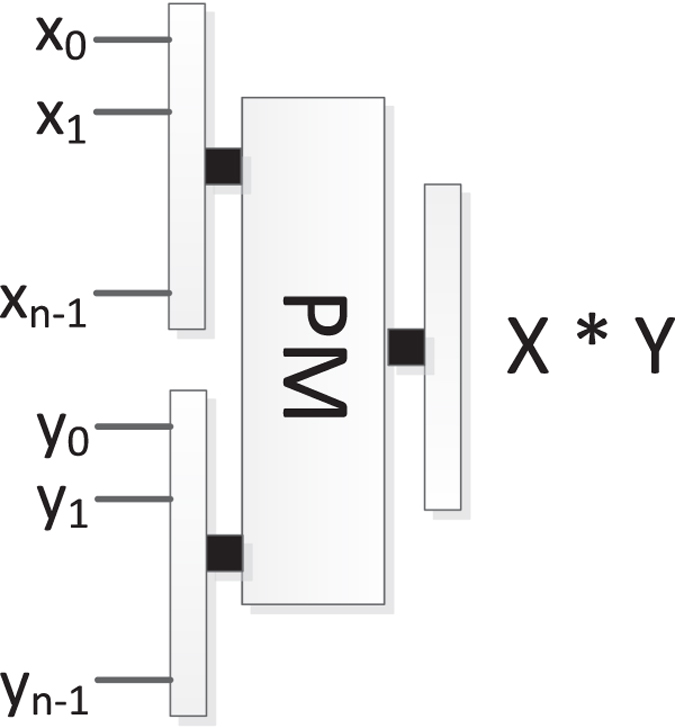
The block diagram of PM.

**Figure 19:**
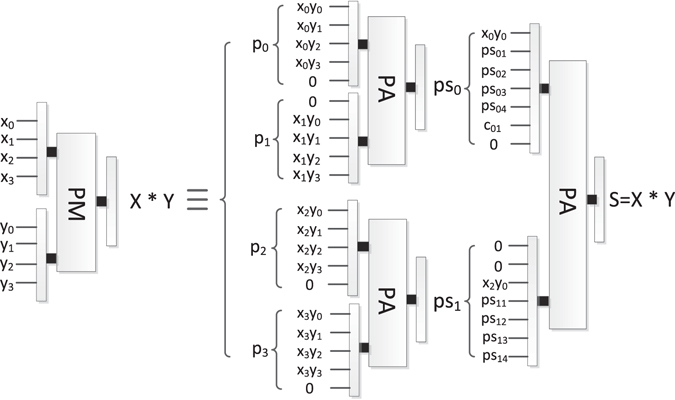
An example of 4 × 4 PM, where *PS*_0_ and *PS*_1_ represent the partial sums of *P*_0_, *P*_1_, *P*_2_, *P*_3_.

Kotiyal S *et al*.^37^ proposed the PM based on the binary tree which optimized the ancillary and garbage bits. The block diagram of PM is shown in Fig. 18, where PM consists of multiple reversible parallel adders. The PM can implement the multiplication *X* × *Y*, where $X={x}_{n-1}\cdots {x}_{1}{x}_{0}$ and $Y={y}_{n-1}\cdots {y}_{1}{y}_{0}$ are the two inputs. An example of 4 × 4 PM is shown in Fig. 19.

### The reversible divider (ND)

**Figure 20:**
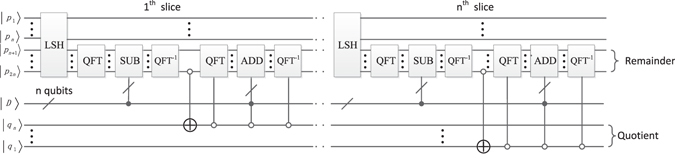
Quantum divider circuit based on restoring division algorithm.

Khosropour A *et al*.^38^ realized quantum division circuit based on restoring division algorithm as shown in Fig. 20.

Here, $|P\rangle =|{P}_{2n-1}\cdot \cdot \cdot {P}_{2}{P}_{1}\rangle$, $|D\rangle =|{D}_{n-1}\cdot \cdot \cdot {D}_{2}{D}_{1}\rangle$, $|Q\rangle =|{Q}_{n-1}\cdot \cdot \cdot {Q}_{2}{Q}_{1}\rangle$ are the input registers. Multiply $|P\rangle$ by 2 can be obtained by the left shift (LSH)^39^ module. For realizing subtracting $|D\rangle$ from the highest *n* qubits of $|2P\rangle$, we first act quantum Fourier transform (QFT)^22^ on the highest *n* qubits of $|2P\rangle$ and apply a set of conditional rotation operations on the *n* qubits of $|D\rangle$. Secondly, we perform inverse QFT (QFT^−1^) and check if $|(2P-D)\rangle$ is either positive or negative. If it is positive, the qubit $|{Q}_{n}\rangle$ need to be initialized into state |0〉, otherwise, keep $|{Q}_{n}\rangle$ unchanged, which can be realized using a CNOT gate. If $|(2P-D)\rangle$ is diagnosed to be negative, it should be set back to the previous state $|2P\rangle$ by simply adding $|D\rangle$ to the highest n qubits of $|2P-D\rangle$. Because $|{Q}_{n}\rangle$ contains the inverse of the most significant qubits in $|2P-D\rangle$. Hence, the addition should be conditioned on $|{Q}_{n}\rangle$. This structure should be repeated *n* times to fulfill the division operation. Eventually, $|Q\rangle$ is the quotient and the highest n qubits of |P〉 is the remainder. Note that we have used only *n* ancillary qubits for LSH operation.

**Figure 21:**
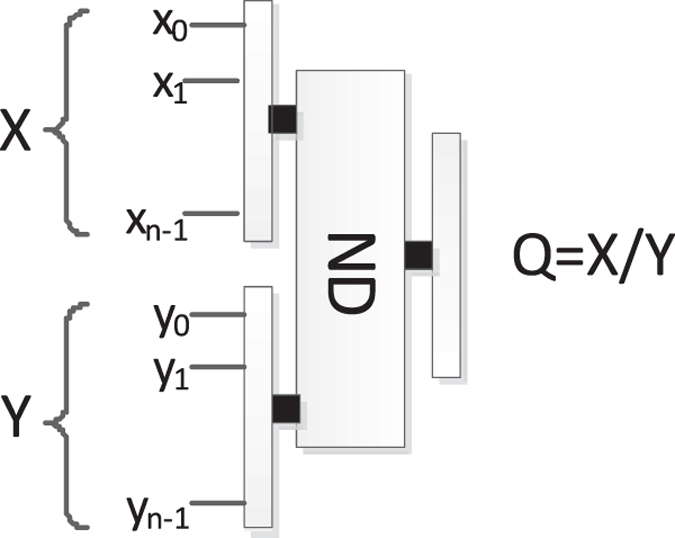
The simplified graph of the quantum division circuit.

For convenience, Fig. 21 is a simplified graph of the quantum division circuit in Fig. 20, where ancillary inputs and garbage outputs are omitted, and Q is the quotient.

### Feasibility and rationality of bilinear interpolation method

In this paper, the quantum circuit of the image scaling based on bilinear interpolation method for NEQR is designed. Therefore, the first problem is to prove the practicality. The key idea of the proposed circuits is mathematically explained in Eq. (7)

7 $$|{C}_{Y^{\prime} X^{\prime} }\rangle =[\begin{matrix}[{2}^{m}-(Y^{\prime} -Y\times {2}^{m})]\times [{2}^{m}-(X^{\prime} -X\times {2}^{m})]\times |{C}_{Y,X}\rangle \\ +(Y^{\prime} -Y\times {2}^{m})\times [{2}^{m}-(X^{\prime} -X\times {2}^{m})]\times |{C}_{Y+1,X}\rangle \\ +[{2}^{m}-(Y^{\prime} -Y\times {2}^{m})]\times (X^{\prime} -X\times {2}^{m})\times |{C}_{Y,X+1}\rangle \\ +(Y^{\prime} -Y\times {2}^{m})\times (X^{\prime} -X\times {2}^{m})\times |{C}_{Y+1,X+1}\rangle \end{matrix}]\div{2}^{m}\div{2}^{m}$$

where $$X=\frac{X^{\prime} }{{2}^{m}},Y=\frac{Y^{\prime} }{{2}^{m}}$$.

From Eq. (7), in order to prepare the color information $|{C}_{Y^{\prime} ,X^{\prime} }\rangle$ in position (*Y*′, *X*′) of the resulting image, the color information $|{C}_{Y,X}\rangle ,|{C}_{Y+1,X}\rangle ,|{C}_{Y,X+1}\rangle$ and $|{C}_{Y+1,X+1}\rangle$ in positions (*Y*, *X*), (*Y* + 1, *X*), (*Y*, *X* + 1) and (*Y* + 1, *X* + 1) of the original image need to be prepared first. The bilinear interpolation method utilizes these four different positions of the original image to map into one position (*Y*′, *X*′) of the resulting image as shown in Fig. 2. The dimension of a given original image is known and all the pixels should have fixed color values. At the same time, the dimension of the resulting image is known under a certain scaling ratio. Therefore, (*Y*′, *X*′) can be considered as the input state when designing quantum circuits. Under the guidance of this key idea, the qualification process is as follows.

**Theorem** The bilinear interpolation method generated by Eq. (7) is rational for a quantum image based on NEQR.

**Proof** Assume that the size of an original quantum image $|{\rm{I}}\rangle$ is 2^*n*^ × 2^*n*^, and the gray range of which is [0, 2^*q*^ − 1], the NEQR of the image is expressed by Eq. (8).

8 $$|I\rangle =\frac{1}{{2}^{n}}\sum _{Y=0}^{{2}^{n}-1}\sum _{X=0}^{{2}^{n}-1}|{C}_{YX}\rangle |Y\rangle |X\rangle =\frac{1}{{2}^{n}}\sum _{YX=0}^{{2}^{2n}-1}\underset{k=0}{\overset{q-1}{\otimes }}{C}_{YX}^{k}\otimes |YX\rangle $$

Also suppose image scaling ratio in the horizontal and vertical dimensions is 2^*m*^, than is *r*_*y*_ = *r*_*x*_ = 2^*m*^, then the size of the resulting image $|{\rm{I}}^{\prime} \rangle$ is 2^*n*+*m*^ × 2^*n*+*m*^. The concrete feasibility of the bilinear interpolation is proven through the following analysis.

**Problem 1** How to build the interpolation mapping relationship between the pixel of the resulting image and the original image?

The position (*Y*′, *X*′) of the resulting image has the mapping relationship with the positions (*Y*, *X*), (*Y* + 1, *X*), (*Y*, *X* + 1) and (*Y* + 1, *X* + 1) of the original image as shown in Fig. 2.

According to Eq. (3), we derive the Eq. (9)

9 $$Y=\lfloor Y^{\prime} \times (\frac{{2}^{n}}{{2}^{n+m}})\rfloor =\lfloor \frac{Y^{\prime} }{{2}^{m}}\rfloor ,X=\lfloor X^{\prime} \times (\frac{{2}^{n}}{{2}^{n+m}})\rfloor =\lfloor \frac{X^{\prime} }{{2}^{m}}\rfloor $$

where $${\rm{Y}}^{\prime} ={Y}_{n+m-1}^{^{\prime} }{Y}_{n+m-2}^{^{\prime} }\ldots {Y}_{1}^{^{\prime} }{Y}_{0}^{^{\prime} }$$ and $${\rm{X}}^{\prime} ={X}_{n+m-1}^{^{\prime} }{X}_{n+{\rm{m}}-{\rm{2}}}^{^{\prime} }\ldots {X}_{1}^{^{\prime} }{X}_{0}^{^{\prime} }$$.

**Figure 22:**
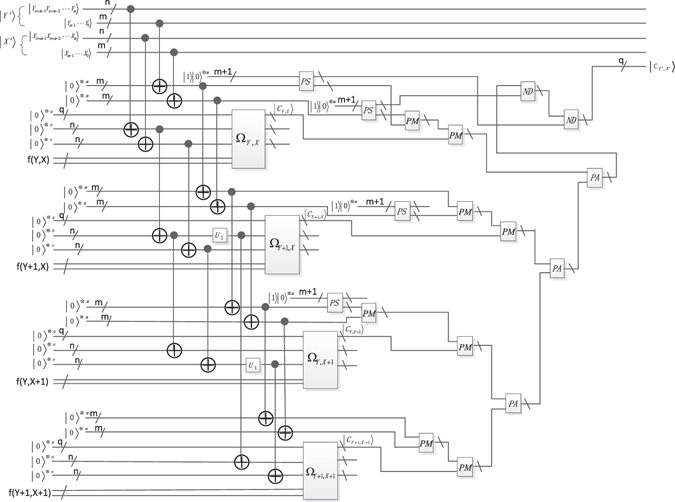
The scaling-up circuit of bilinear interpolation method for NEQR.

**Figure 23:**
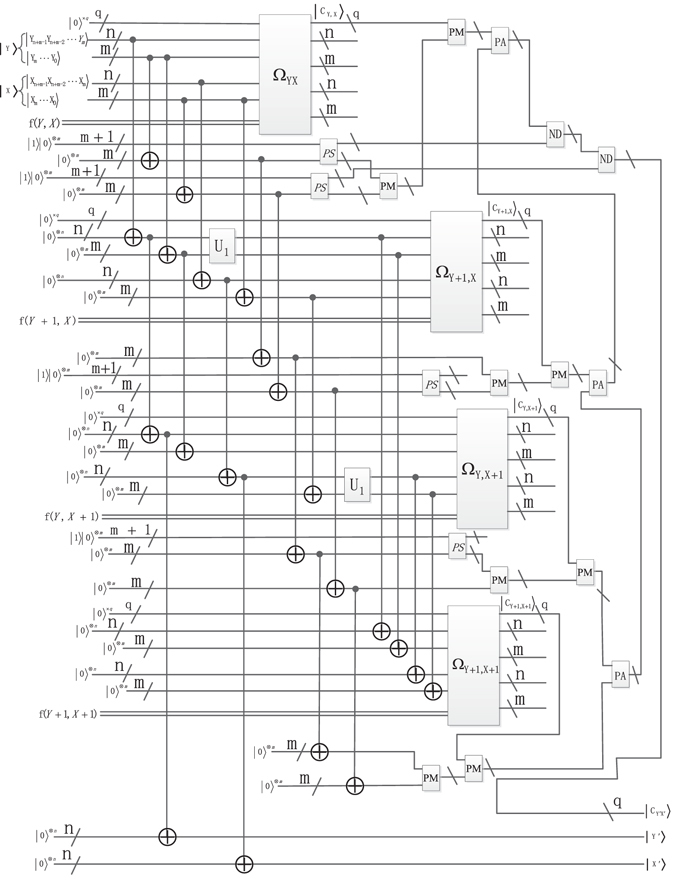
The scaling-down circuit of bilinear interpolation method for NEQR.

To build the mapping relationship described in Fig. 2, the multiply Control-Not operations and special adding one operation ${{\rm{U}}}_{1}({\rm{n}})$ are chosen as the unitary operators. The function of the multiply Control-Not operators is to utilize *n* Control-Not gates to copy the *n* qubits $|{{\rm{Y}}}_{n+m-1}^{^{\prime} }{Y}_{n+m-2}^{^{\prime} }{Y}_{m}^{^{\prime} }\rangle$ into the *n* ancillary qubits ${|0\rangle }^{\otimes n}$. The unitary operator U_1_(*n*) is used to get the nearest-neighbor position of the current position. Through these two unitary operators, the interpolation mapping relationship between the position of original image and the interpolated image has been established. The details are described in Figs 22 and 23.

**Problem 2** How to calculate the color values of the resulting image?

The color information $|{C}_{Y^{\prime} ,X^{\prime} }\rangle$ of the position (*Y*′, *X*′) in the interpolated image is calculated by the four pixels value in position (*Y*, *X*), (*Y* + 1, *X*), (*Y*, *X* + 1) and (*Y* + 1, *X* + 1) of the original image. The gray range $|{C}_{Y^{\prime} ,X^{\prime} }\rangle$ of the interpolated imageis [0, 2^*q*^ − 1], therefore, *q* qubits are needed to store the pixels value of $|{C}_{Y^{\prime} ,X^{\prime} }\rangle$.

Firstly, four quantum oracle operators Ω_*Y*,*X*_, Ω_*Y*+1,*X*_, Ω_*Y*,*X*+1_ and Ω_*Y*+1,*X*+1_ are used to compute the original pixel values of $|{C}_{Y,X}\rangle ,|{C}_{Y+1,X}\rangle ,|{C}_{Y,X+1}\rangle$ and $|{C}_{Y+1,X+1}\rangle$, respectively. A quantum oracle operator Ω_*Y*,*X*_ can realize the aim of assigning color information $|{C}_{Y,X}\rangle$ to the ancillary qubits ${|0\rangle }^{\otimes q}$ ^8^, which can be expressed by Eq. (10)

10 $${{\rm{\Omega }}}_{Y,X}{|0\rangle }^{\otimes q}=\underset{i=0}{\overset{q-1}{\otimes }}({{\rm{\Omega }}}_{Y,X}^{i}|0\rangle )=\underset{i=0}{\overset{q-1}{\otimes }}|0\oplus {C}_{Y,X}^{i}\rangle =\underset{i=0}{\overset{q-1}{\otimes }}|{C}_{Y,X}^{i}\rangle =|{C}_{Y,X}\rangle $$

where ${{\rm{\Omega }}}_{Y,X}^{i},i=0,\mathrm{..},q-1$ can be described as the following.

If ${C}_{Y,X}^{i}=1$, ${{\rm{\Omega }}}_{Y,X}^{i}$ is a *2n*-Control-Not qubit gate. Otherwise, it is a quantum identity gate. That is to say, every oracle operator ${{\rm{\Omega }}}_{Y,X}^{i},i=0,\mathrm{..},q-1$ is at most a *2n*-Control-Not qubit gate. For other three oracle operators Ω_*Y*+1,*X*_, Ω_*Y*,*X*+1_, Ω_*Y*+1,*X*+1_, the principle is also same as Ω_*Y*,*X*_^8^.

Therefore, we can calculate each pixel value in position (*Y*′, *X*′) of the resulting image using the pixel values of the four corresponding positions in the original image. From Eq. (3), Eq. (6) and Eq. (9), we can calculate the interpolated pixel value $|{{\rm{C}}}_{{\rm{Y}}^{\prime} ,{\rm{X}}^{\prime} }\rangle$ described by the Eq. (11).

11 $$\begin{matrix}|{C}_{Y^{\prime} X^{\prime} }\rangle  & = & (1-h)(1-w)|{C}_{Y,X}\rangle +h(1-w)|{C}_{Y+1,X}\rangle +(1-h)w|{C}_{Y,X+1}\rangle +hw|{C}_{Y+1,X+1}\rangle \\  & = & [(\begin{matrix}(1-h)(1-w)|{C}_{Y,X}\rangle +h(1-w)|{C}_{Y+1,X}\rangle \\ +(1-h)w|{C}_{Y,X+1}\rangle +hw|{C}_{Y+1,X+1}\rangle \end{matrix})\times {2}^{m}\times {2}^{m}]\div{2}^{m}\div{2}^{m}\\  & = & (\begin{matrix}({2}^{m}-h\times {2}^{m})({2}^{m}-w\times {2}^{m})|{C}_{Y,X}\rangle +h\times {2}^{m}({2}^{m}-w\times {2}^{m})|{C}_{Y+1,X}\rangle \\ +({2}^{m}-h\times {2}^{m})w\times {2}^{m}|{C}_{Y,X+1}\rangle +h\times {2}^{m}\times w\times {2}^{m}|{C}_{Y+1,X+1}\rangle \end{matrix})\div{2}^{m}\div{2}^{m}\\  & = & [\begin{matrix}[{2}^{m}-(Y^{\prime} -Y\times {2}^{m})]\times [{2}^{m}-(X^{\prime} -X\times {2}^{m})]\times |{C}_{Y,X}\rangle \\ +(Y^{\prime} -Y\times {2}^{m})\times [{2}^{m}-(X^{\prime} -X\times {2}^{m})]\times |{C}_{Y+1,X}\rangle \\ +[{2}^{m}-(Y^{\prime} -Y\times {2}^{m})]\times (X^{\prime} -X\times {2}^{m})\times |{C}_{Y,X+1}\rangle \\ +(Y^{\prime} -Y\times {2}^{m})\times (X^{\prime} -X\times {2}^{m})\times |{C}_{Y+1,X+1}\rangle \end{matrix}]\div{2}^{m}\div{2}^{m}\end{matrix}$$

We need implement some arithmetic operations to calculate the pixel value $|{C}_{Y^{\prime} ,X^{\prime} }\rangle$. According to Eq. (9), *Y*′ − *Y* × 2^*m*^ and *X*′ − *X* × 2^*m*^ are the remainder of $\frac{Y^{\prime} }{{2}^{m}}={y}_{m-1}{y}_{m-2}\cdots {y}_{0}$ and $\frac{X^{\prime} }{{2}^{m}}={x}_{m-1}{x}_{m-2}\cdots {x}_{0}$. The *m* Control-Not operations are used to copy the remainder into the *m* qubits ${|0\rangle }^{\otimes m}$. Other arithmetic operations such as subtraction, multiplication, addition and division are implemented by corresponding arithmetic circuits mentioned in the preceding chapter. Hence, the pixel value of $|{C}_{Y^{\prime} ,X^{\prime} }\rangle$ can be derived through the analysis above, the concrete circuit is shown in Figs 22 and 23.

### Quantum realization of the bilinear interpolation method

In the previous section, the theoretical feasibility of the bilinear interpolation method for NEQR is discussed using the multiply Control-Not operation, special adding one operation and a series of quantum circuit modules. This section gives the concrete quantum realization circuit of the bilinear interpolation method for NEQR, including scaling up and scaling down.

### Quantum image scaling up circuit of the bilinear interpolation for NEQR

Assume that a 2^*n*^ × 2^*n*^ quantum image $|I\rangle$ is scaled up to a 2^*n*+*m*^ × 2^*n*+*m*^ quantum image $|I^{\prime} \rangle$ based on the bilinear interpolation. The scale ratio in the vertical and horizontal level is both 2^*m*^, that is to say, *r*_*y*_ = *r*_*x*_ = 2^*m*^.

### The concrete scaling-up circuit for NEQR

Figure 22 provides the quantum image scaling-up circuit that implements the bilinear interpolation for NEQR. The concrete steps can be described as follows.

**Step 1** Firstly, obtain the $Y=\frac{Y^{\prime} }{{2}^{m}}$ using *n* Control-Not gates on the coordinate $|{{\rm{Y}}}_{n+m-1}{Y}_{n+m-2}\cdot \cdot \cdot {Y}_{m}\rangle$. Correspondingly, obtain the ${\rm{X}}=\frac{X^{\prime} }{{2}^{m}}$ through *n* Control-Not gates on the coordinate $|{{\rm{X}}}_{n+m-1}{X}_{n+m-2}\cdots {X}_{m}\rangle$. Then, obtain the positions (*Y* + 1, *X*), (*Y*, *X* + 1) and (*Y* + 1, *X* + 1) using *6n* Control-Not gates and two special adding one operators. Therefore, the coordinate mapping has been built between the position (*Y*′, *X*′) in resulting image and the positions (*Y*, *X*), (*Y* + 1, *X*), (*Y*, *X* + 1) and (*Y* + 1, *X* + 1) in original image. In addition, 4 (*m* + 1)-ancillary constant qubits $|1\rangle {|0\rangle }^{\otimes m}$ and 8 *m* Control-Not gates are needed to copy the qubits $|{Y}_{m-1}\cdots {Y}_{0}\rangle$ and qubits $|{X}_{m-1}\cdots {X}_{0}\rangle$ to 8 *m* ancillary qubits ${|0\rangle }^{\otimes m}$.

**Step 2** Employ four oracle operators Ω_*Y*,*X*_, Ω_*Y*+1,*X*_, Ω_*Y*,*X*+1_ and Ω_*Y*+1,*X*+1_ to generate the corresponding color information $|{C}_{Y,X}\rangle ,|{C}_{Y+1,X}\rangle ,|{C}_{Y,X+1}\rangle$ and $|{C}_{Y+1,X+1}\rangle$.

**Step 3** Calculate the pixel value $|{C}_{Y^{\prime} ,X^{\prime} }\rangle$ of the interpolated image through the four pixel values $|{C}_{Y,X}\rangle ,|{C}_{Y+1,X}\rangle ,|{C}_{Y,X+1}\rangle$ and $|{C}_{Y+1,X+1}\rangle$ as described in Eq. (10). The realization circuit is shown in Fig. 22.

### Circuit complexity

The circuit network complexity depends on the number of the elementary gate in QIMP. The complexity of the basic quantum gate is considered to be 1 including NOT gate, Control-Not gate and any 2 × 2 unitary operator^40^. In addition, when designing the quantum circuit, introducing ancillary qubit $|{\rm{0}}\rangle$ or $|{\rm{1}}\rangle$ is a commonly used method. The complexity of Fig. 22 is analyzed as follows.

In step 1, it needs 8(*n* + *m*) Control-Not gates and two U_1_(*n*) operators. As shown in Fig. 10, each unitary operator U_1_(*n*) has *n* − 1 Not gates, *n* + 1 Control-Not gates, and *n* − 1 multi-Control-Not gates of ${{\rm{\Lambda }}}_{2}({\sigma }_{x}),\cdot \cdot \cdot ,{{\rm{\Lambda }}}_{n-1}({\sigma }_{x}),{{\rm{\Lambda }}}_{n}({\sigma }_{x})$ and one (*n* − 1)-Control-Not gate, where *σ*_*x*_ is the NOT gate. According to Lemma 6.1 and Lemma 7.1 in ref. 40, for any 2 × 2 unitary matrix U and any *n* ≥ 3, a Λ_*n*−1_(*U*) gate can be simulated by *n* qubits circuit consisting of (2^*n*−1^ − 1)Λ_1_(*V*) gates, a Λ_1_(*V* ^+^) gate and (2^*n*−1^ − 2)Λ_1_(*σ*_*x*_) gates, where *V* represents the unitary operator, *V* ^+^ is the hermitian conjugate of *V*. We can deduce that the network complexity of single quantum operator U_1_(*n*) is Ο(2^*n*+2^). Thus, the total quantum cost in this step is Ο(2^*n*+3^ + 8*n* + 8*m*).

In step 2, it includes four oracle operators of Ω_*Y*,*X*_, Ω_*Y*+1, *X*_, Ω_*Y*,*X*+1_ and Ω_*Y*+1,*X*+1_. The complexity of oracle operator is Ο(*q* ⋅ 2*n*)^8^, therefore, the total quantum cost in this step is Ο(*q* ⋅ 8*n*).

In step 3, it includes 4 parallel subtractors, 8 parallel multipliers, 3 parallel adders, 2 reversible dividers.

#### The network complexity of PS

The PS realizes the subtraction between two *m* + 1 qubits. It needs 1 RHS and *m* reversible full subtractors. The quantum cost of RHS (see Fig. 15) is 6, and the quantum cost of RFS (see Fig. 16) is 7. So the quantum cost of single PS is 7 *m* + 6.

#### The quantum cost of PA

As we know, the pixel value is represented by *q* qubits, the resulting pixel value can be calculated by implementing ND modules twice. Thus, the three parallel adders perform the operation of *q* + 2*m* + 2 qubits plus *q* + 2*m* + 2 qubits, it needs 1 RHA and (*q* + 2 *m* + 1) reversible full adders. The quantum cost of RHA (see Fig. 12) is 4, and the quantum cost of RFA (see Fig. 13) is 8. Therefore, the quantum cost of single PA is 8*q* + 16 *m* + 20.

#### The network complexity of PM

As shown Fig. 22, it is easy to find there are 4 parallel multipliers, each of which performs *m* + 1 qubits multiply by *m* + 1 qubits. We can see form Fig. 19 that each PA can add two *m* + *1* qubits at most. Thus, the number of PA required in PM is ${\rm{1}}+2+\cdots +\frac{m+1}{2}$.

In another case, the other 4 parallel multipliers perform 2 *m* + *2* qubits multiply by *q* qubits respectively (suppose q ≥ 2*m* + 2). We can see form Fig. 19 that each PA can add two 2(*m* + *1*) qubits at most. Thus, the number of PA required in PM is ${\rm{1}}+2+\cdots +\frac{2m+2}{2}$.

Thus, we can calculate that the network complexity of PM is

$$4\times [\begin{matrix}(5{(m+1)}^{2}+(1+2+\cdot \cdot \cdot \frac{m+1}{2})(16m+12)\\ +5\cdot q(2m+2)+(1+2+\cdot \cdot \cdot \frac{2m+2}{2})(8q+16m+20)\end{matrix}]$$

#### The network complexity of ND

According to paper^38^, the quantum cost of *q*-qubit ND is 3*q* ^3^ + 6*q* ^2^ + *q*.

Consequently, the complexity of the proposed scaling-up circuit shown in Fig. 22 is calculated by Eq. (12).

12 $$\begin{matrix}O(\begin{matrix}{2}^{n+3}+8n+8m+8qn+4(7m+6)+3(8q+16m+20)\\ +2(3{(q+2m+2)}^{3}+2{(q+2m+2)}^{2}+q+2m+2)\\ +4(5{(m+1)}^{2}+(1+2+\cdots +\frac{m+1}{2})(16m+12))\\ +4(5q(2m+2)+(1+2+\cdots +\frac{2m+2}{2})(8q+16m+20))\end{matrix})\\ \,\approx O({2}^{n+3})\end{matrix}$$

### Quantum image scaling-down circuit of the bilinear interpolation for NEQR

Assume that a 2^*n*+*m*^ × 2^*n*+*m*^ quantum image $|I\rangle$ is scaled down to a 2^*n*^ × 2^*n*^ quantum image $|I^{\prime} \rangle$ based on bilinear interpolation. The scale ratio is $\frac{1}{{2}^{m}}$ whether in the vertical or horizontal level, that is to say ${r}_{y}={r}_{x}={2}^{-m}=\frac{1}{{2}^{m}}$.

### Concrete circuit for NEQR

Fig. 23 shows the quantum image scaling-up circuit that implements the bilinear interpolation for NEQR. The concrete steps are described as follows.

**Step 1** First of all, obtain the positions of (*Y* + 1, *X*), (*Y*, *X* + 1) and (*Y* + 1, *X* + 1) using 6(*n* + *m*) Control-Not gates and two special adding one *U*_1_(*n* + *m*) operators. Then, obtain the $Y=\frac{Y^{\prime} }{{2}^{m}}$ acting *n* Control-Not gates on the coordinate $|{{\rm{Y}}}_{n+m-1}{Y}_{n+m-2}\cdots {Y}_{m}\rangle$. Correspondingly, obtain the ${\rm{X}}=\frac{X^{\prime} }{{2}^{m}}$ through *n* Control-Not gates on the coordinate $|{{\rm{X}}}_{n+m-1}{X}_{n+m-2}\cdots {X}_{m}\rangle$. Therefore, the coordinates mapping relationship has been built between the position (*Y*′, *X*′) of the resulting image and the positions in (*Y*, *X*), (*Y* + 1, *X*), (*Y*, *X* + 1) and (*Y* + 1, *X* + 1) of the original image.

**Step 2** Employ four oracle operators Ω_*Y*,*X*_, Ω_*Y*+1,*X*_, Ω_*Y*,*X*+1_ and Ω_*Y*+1,*X*+1_ to generate the color information $|{C}_{Y,X}\rangle ,|{C}_{Y+1,X}\rangle ,|{C}_{Y,X+1}\rangle$ and $|{C}_{Y+1,X+1}\rangle$.

**Step 3** Calculate pixel value $|{C}_{Y^{\prime} ,X^{\prime} }\rangle$ of the interpolated image through the four pixel values of $|{C}_{Y,X}\rangle ,|{C}_{Y+1,X}\rangle ,|{C}_{Y,X+1}\rangle$ and $|{C}_{Y+1,X+1}\rangle$ described in Eq. (10). The concrete realization circuit is shown in Fig. 23.

### Circuit complexity

In step 1, it needs 8*n* + 14 *m* Control-Not gates and 2 *U*_1_(*n* + *m*) operators. Based on the analysis above, the complexity of a single quantum operator *U*_1_(*n* + *m*) is Ο(2^*n*+*m*+2^). Thus, the total network complexity in this step is Ο(2^*n*+*m*+3^ + 8*n* + 14*m*).

In step 2, it includes four oracle operators of Ω_*Y*,*X*_, Ω_*Y*+1,*X*_, Ω_*Y*,*X*+1_ and Ω_*Y*+1,*X*+1_. Then, the total network complexity in this step is ${\rm O}(q\cdot 8(n+m))$.

In step 3, it includes 4 parallel subtractors, 8 parallel multipliers, 3 parallel adders and 2 reversible dividers.

Consequently, the network complexity of the proposed scaling-up circuit shown in Fig. 23 is

$$\begin{matrix}O(\begin{matrix}{2}^{n+m+3}+8n+14m+8q(n+m)+4(7m+6)+3(8q+16m+20)\\ +2(3{(q+2m+2)}^{3}+2{(q+2m+2)}^{2}+q+2m+2)\\ +4(5{(m+1)}^{2}+(1+2+\cdots +\frac{m+1}{2})(16m+12))\\ +4(5q(2m+2)+(1+2+\cdots +\frac{2m+2}{2})(8q+16m+20))\end{matrix})\\ \,\approx O({2}^{n+m+3})\end{matrix}$$

### Simulation experiments and analysis

In this section, the simulation experiments are performed to show the interpolation results. All experiments are simulated by MATLAB sofeware.

### Simulation results of interpolation for NEQR

**Figure 24:**
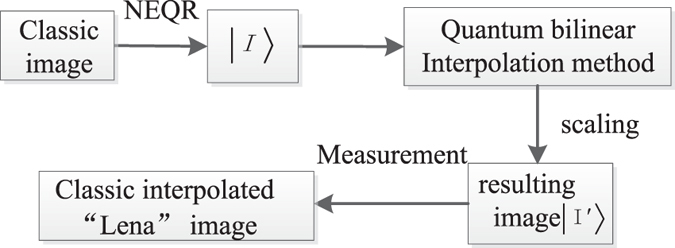
The concrete procedure of quantum bilinear interpolation method.

Fig. 24 gives a concrete procedure of quantum bilinear interpolation. Firstly, we need to transform a classic image into a quantum image $|I\rangle$ expressed by NEQR. Then, the quantum image $|I\rangle$ acts as the input image. The resulting image (the interpolated image) $|I^{\prime} \rangle$ can be derived using the proposed quantum bilinear interpolation method. Finally, we can retrieve the interpolated classic image by quantum measurement.

**Figure 25:**
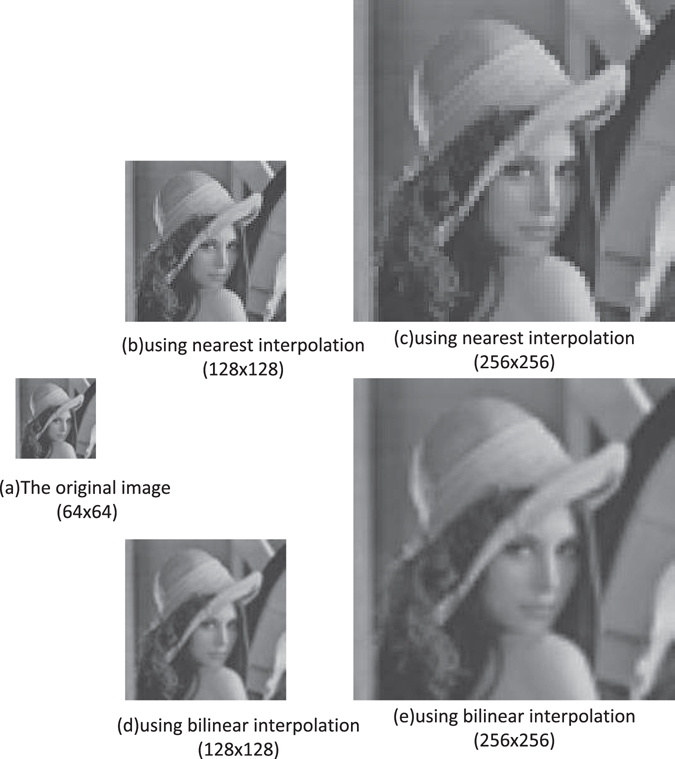
The simulation results using different interpolation method.

The simulation results using different interpolation method are shown in Fig. 25. Figure 25(a) is a 64 × 64 original image named Lena. Figs 25(b,c) are the corresponding 128 × 128 scaling-up NEQR images using nearest-neighbor interpolation and bilinear interpolation, respectively. The scaling ratio is *r*_*x*_ = *r*_*y*_ = 2, which means *n* = 6, *m* = 1 and *q* = 8. Figure 25(d,e) are the corresponding 256 × 256 scaling-up NEQR images using nearest-neighbor interpolation and bilinear interpolation, respectively. The scaling ratio is *r*_*x*_ = *r*_*y*_ = 4, which means *n* = 6, *m* = 2 and *q* = 8. The simulation results indicate that the scaled-up image using bilinear interpolation is clearer than nearest-neighbor interpolation.

### Quantum measurement of the interpolated image

An interpolated NEQR image can be described as following.

$$|I^{\prime} \rangle =\frac{1}{{2}^{n+m}}\sum _{Y^{\prime} =0}^{{2}^{n+m}-1}\sum _{X^{\prime} =0}^{{2}^{n+m}-1}|{C}_{Y^{\prime} X^{\prime} }\rangle \otimes |Y^{\prime} \rangle |X^{\prime} \rangle $$

Obviously, an interpolated NEQR image is a quantum superposition state, which can be regarded as a composite quantum system composed of 2*n* + *q* qubits.

**Figure 26:**
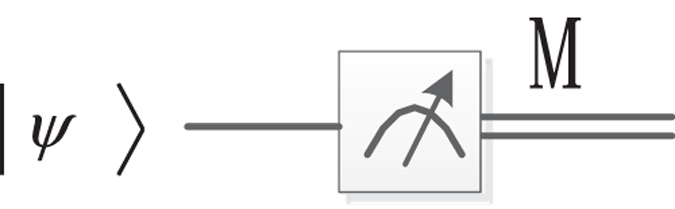
Quantum circuit symbol for measurement.

Actually, the quantum state cannot be practically observed in quantum system because a measurement will destroy the superposition. What is worse, it is not allowed to make copies of the state and measure each one due to the non-cloning theorem. Hence, it is necessary to repeat constructing the states of interpolated image *n* (*n* > 1) times and measure each state to summarize the measurement results, through which we can estimate the interpolated image. We execute probability measurement on the interpolated image. Probability measurement converts the quantum information into classical information in form of probability distributions, i.e., it converts a single qubit state $|\psi \rangle =\alpha |0\rangle +\beta |1\rangle$ into a probability classical bit *M* (distinguished from a qubit by drawing it as a double-line wire), which is 0 with probability *α* ^2^ or 1 with probability *β* ^2^, as shown in Fig. 26.

**Figure 27:**
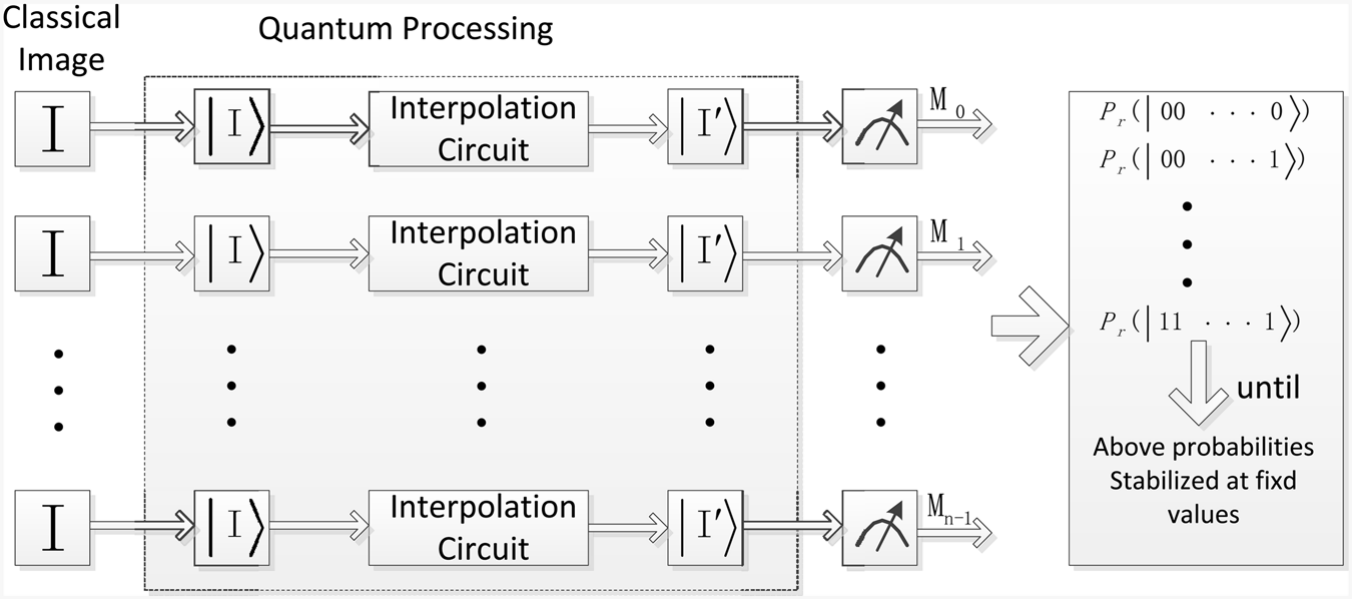
The block diagram of the measurement procedure on quantum computers.

Next, we analyze the impact of quantum measurements on the interpolated image. The measurement results of the 2^*n*+*m*^ × 2^*n*+*m*^ interpolated NEQR image with gray range [0, 2^*q*−1^] are some collection of basis states $\{{S}_{1},{S}_{2},\mathrm{...},{S}_{2(n+m)+q}\}$. After multiple measurements, these basis states follow a probability distribution. The measurement will continue until the probability of each basis state is stabilized at a fixed value. According to law of large numbers, there is a limit to these basis states which can be used to estimate the color information of the interpolated image. The block diagram of the measurement procedure on quantum computers is shown in Fig. 27.

## Conclusions

In this paper, the bilinear interpolation method for NEQR is proposed for the first time. The proposed method constructs an interpolated image, which mainly consists of two steps: (1) position mapping (2) calculate and generate the new color information. In the position mapping stage, the multiply Control-Not operation and special adding one operation are used to build the position mapping relationship between the position (*Y*′, *X*′) in interpolated image and the positions (*Y*, *X*), (*Y* + 1, *X*), (*Y*, *X* + 1) and (*Y* + 1, *X* + 1) in the original image. After that, exploit the oracle operator to prepare the original image pixel. Then, a series of quantum circuits designed in this paper are used to calculate the color information of the interpolated image.

The main contributions of this paper are as follows:

1. The bilinear interpolation method for NEQR is realized and the corresponding quantum realization circuits are given.
2. A series of unitary quantum circuit operations are designed, which can be used in future quantum computers.
3. The quantum image scaling algorithm is developed to change the image size.

The future works mainly include:

1. Give the bilinear interpolation method for FRQI and its realization circuit.
2. Further realize the bicubic interpolation method for other quantum image representations such as FRQI and NEQR.
3. Give simpler quantum interpolation realization circuit through the basic quantum gates and the quantitative analysis about the circuit complexity.
